# Useful Memorials

**Published:** 1888-12

**Authors:** 


### USEFUL MEMORIALS.

Hospital endowments have long been a favorite form of memorial, and, when properly conducted, have served a most useful purpose, for such hospitals constitute a very practical charity. Within the last few years, New York City has been especially favored in this way.

The growth within recent years of training-schools for nurses has led to such improvement in nursing, and the advantages of it have been so marked and so much appreciated, both by the medical profession and the public, that encouragement is properly being given to increase the number of such schools, and to extend the field of usefulness of their graduates. This work not only renders the advantages of hospitals greater, by increasing their efficiency, but its beneficial effect is manifest outside of them. Most of the training-schools established in this country have been organized for the education of women for the duties of a nurse, but recently a school has been endowed in New York for the education of men, recognizing the fact that there are cases in which men are better adapted for some special duties in nursing, and that they should have an opportunity to properly qualify themselves for that work.

Another philanthropic gentleman has just completed a memorial training-school, called *The Henry W. Bishop, 3d, Memorial Training School for Nurses*, at Pittsfield, Massachusetts. It was built by Henry W. Bishop, of Chicago, as a useful memorial of his son, and in evidence of his appreciation of the value of the services rendered by trained nurses to his son during his fatal illness at Pittsfield, and, also, with a desire to aid in extending the widening field of usefulness of trained nurses. Such memorials are practical, benevolent, and worthy of commendation.

